# Investigating the Presence of SARS CoV-2 in Free-Living and Captive Animals

**DOI:** 10.3390/pathogens10060635

**Published:** 2021-05-21

**Authors:** Lorena Jemeršić, Ivana Lojkić, Nina Krešić, Tomislav Keros, Tajana Amšel Zelenika, Luka Jurinović, Damir Skok, Ingeborg Bata, Jadranko Boras, Boris Habrun, Dragan Brnić

**Affiliations:** 1 Virology Department, Croatian Veterinary Institute, Savska Cesta 143, 10000 Zagreb, Croatia; lemo@veinst.hr (N.K.); keros@veinst.hr (T.K.); habrun@veinst.hr (B.H.); brnic@veinst.hr (D.B.); 2 Poultry Center, Croatian Veterinary Institute, Heinzelova 55, 10000 Zagreb, Croatia; t_amsel-zelenika@veinst.hr (T.A.Z.); jurinovic@veinst.hr (L.J.); 3 Zagreb Zoo, Maksimirski Perivoj, 10000 Zagreb, Croatia; damir.skok@zoo.hr (D.S.); inga.bata@zoo.hr (I.B.); jadranko.boras@zoo.hr (J.B.)

**Keywords:** SARS CoV-2, serology, RNA detection, free-living wild animals, zoo animals

## Abstract

Due to SARS CoV-2 recombination rates, number of infected people and recent reports of environmental contamination, the possibility of SARS CoV-2 transmission to animals can be expected. We tested samples of dominant free-living and captive wildlife species in Croatia for the presence of anti-SARS CoV-2 antibodies and viral RNA. In total, from June 2020 until February 2021, we tested blood, muscle extract and fecal samples of 422 free-living wild boars (*Sus scrofa*), red foxes (*Vulpes vulpes*) and jackals (*Canis aureus*); blood and cloacal swabs of 111 yellow-legged gulls (*Larus michahellis*) and fecal samples of 32 zoo animals. A commercially available ELISA (ID.Vet, France) and as a confirmatory test, a surrogate virus neutralization test (sVNT; GenScript, Netherlands) were used. Fecal samples were tested for the presence of viral RNA by a real-time RT–PCR protocol. Fifteen out of 533 (2.8%) positive ELISA results were detected; in wild boars (3.9%), red foxes (2.9%) and jackals (4.6%). However, the positive findings were not confirmed by sVNT. No viral RNA was found. In conclusion, no spillover occurred within the investigated period (second COVID-19 wave). However, further investigation is needed, especially regarding wildlife sample features for serological tests.

## 1. Introduction

Coronaviruses belong to the virus family Coronaviridae, subfamily Orthocoronavirinae that includes four genera: alpha-, beta-, gamma- and deltacoronavirus. The natural hosts of alpha- and betacoronaviruses are bats, while birds are common reservoirs of gamma- and deltacoronaviruses [1,2]. Even so, many coronaviruses belonging to all four genera are causative agents of respiratory, gastrointestinal or neurological infections in humans, swine, horses, cattle, camels, cats, dogs, rodents, water mammals, minks, ferrets, birds and other wild animals [3].

The causative agent of COVID-19, the severe acute respiratory syndrome coronavirus 2 (SARS CoV-2), is a betacoronavirus, comprising one of the largest (27–34 kb) single-stranded, positive-sense RNA molecules of all known viruses. Two-thirds of the molecule contains an open reading frame (ORF) that encodes a replicase/transcriptase polyprotein, post-translationally cleaved into smaller nonstructural proteins engaged in viral replication. The remaining ORF (10 kb) encodes four structural proteins, the membrane (M), envelope (E), spike (S) and nucleocapsid (N) proteins [4]. The N and S proteins induce the development of specific antibodies [5,6,7,8,9]. The S-protein also attaches to the complementary protein of the host cell, defining the cell tropism of a coronavirus. Most of the known betacoronaviruses, including SARS CoV-2, attach to the angiotensin-converting enzyme 2 (ACE2) receptor-binding domains, located on the surface of cells primarily in the lungs, but also in the arteries, heart, kidney, and intestines [10,11]. In the past two decades, novel coronavirus strains of higher virulence and pathogenicity were reported as causes of epidemic outbreaks in both humans and animals. The most important public health coronavirus infections before COVID-19 were the severe acute respiratory syndrome, SARS [12] and the Middle-East respiratory syndrome, MERS [13]. Both are caused by betacoronaviruses transmitted from bats to humans by an intermediate animal host, the Asian palm civet (*Paradoxurus hermaphrodites*) and the Arabian camel (*Camelus dromedarius*), respectively [14].

Since the beginning of the COVID-19 pandemic in 2019, it is presumed that the infection emerged from a betacoronavirus circulating in Rhinolophidae (horseshoe) bats [15,16]. However, the potential intermediate host of SARS CoV-2 is still unknown. Various animal species, such as snakes and pangolins, have been investigated [17,18,19,20], with no consensus being reached. As of 6 April 2021, the World Organization for Animal Health (OIE) had recorded 458 cases of SARS CoV-2 infections in animals defined as positive SARS CoV-2 RNA findings or a result of SARS CoV-2 isolation from one or more animals [21].

Until today, SARS CoV-2 is detected in naturally infected felines (tiger, lion, pet cats, snow leopard and a puma), dogs, a ferret, wild mink (*Neovison vison*), farm minks and a gorilla [22,23,24,25]. Infections in animals are typically asymptomatic, while mild clinical signs of a self-limiting, respiratory and/or gastrointestinal disease are found in felines. A severe to fatal course of infection is described only in minks [25]. The first known infection by SARS CoV-2 in farm animals is described in a Dutch mink farm. The genetic analyses of strains derived from minks and infected farm workers suggest a spillover from humans to minks resulting in a possible spillback event from minks to humans [25]. Since then, cases of SARS CoV-2 infection in mink farms are recorded in seven European countries, the USA and Canada, and new SARS CoV-2 mink-variants are recognized, indicating possible interspecies transmission from minks to in-contact people [26]. The detection of SARS CoV-2 RNA in a non-captive wild mink [27] highlights the need for targeted monitoring of susceptible wildlife species.

To identify an intermediate host or potential reservoirs of SARS CoV-2, assessments of different animal species likelihood of susceptibility towards SARS-CoV-2 based on the comparative analyses of coding sequences and amino acid structures of its receptor-binding domain (RBD) affinity within ACE2 and transmembrane protease, serine 2 (TMPRSS2) were carried out. The results show that non-human primates are of relatively high potential risk [28,29], followed by carnivores, cetaceans (aquatic mammals) and wild rodents [29]. In contrast, rodents that were experimentally infected by SARS CoV-2 showed lower risk [29,30]. Birds, reptiles and amphibians occupied the lowest ranges in the analyses. Another simulation model classified domestic cats, tigers and golden hamsters to be only of medium risk and ferrets of low-risk [28]; however, the incidence of natural infections in these animals has shown to be the highest. Pigs, dogs, cattle and sheep were considered resistant to SARS CoV-2 due to the results of an experimental trial [23]. Nevertheless, ACE2 proteins capable of acting as receptors for viral cell entry are discovered in the species mentioned above, hypothetically allowing infection [31,32]. Therefore, different simulation models showed controversial results related not only to animal species and their potential risk of being susceptible to SARS CoV-2 infections but even regarding the infection pathway (natural or experimental), suggesting that models as predictive tools have limitations [29,30]. Deng et al. (2020) carried out a serological survey of SARS CoV-2 in experimental, domestic, companion and wild animals in China to recognize potential intermediate hosts of SARS CoV-2. The survey was conducted on 35 different animal species, including foxes, a wild boar and a jackal. No sign of natural infection or seroconversion was found in any of the involved species [33]. However, based on in vitro and in vivo experiments, the recognition of susceptible animal species as potential hosts and reservoirs of SARS CoV-2 is not only dependent on the presence of adequate cell receptors but also relies on other factors, such as animal body temperature, population density and group-living of animals as well as the possibility of human to animal direct or indirect contact [34].

Since the main transmission route of SARS CoV-2 is human-to-human, and the number of SARS CoV-2-positive animals is very low, OIE considers animals of low risk for the transmission of SARS CoV-2. However, spillover events from animals to humans, farm animals to wild animals and vice versa have been previously recorded, as recently in influenza, Ebola, Crimean Congo hemorrhagic fever or hepatitis E [35,36,37]. Spillovers may be devastating for different, especially protected animal species and can establish viral reservoirs in the environment threatening public health by the reintroduction of SARS CoV-2 continuously. Due to the number of infected people, possible animal susceptibility, high viral recombination rates of coronaviruses and recent reports of environmental, especially wastewater contamination, reverse spillovers of SARS CoV-2 are inevitable in the future [30,38,39,40].

Members of the Suidae family are often recognized as viral “incubators” [41] and are hosts of some coronaviruses, primarily alphacoronaviruses. Carnivores are considered to be at high risk for SARS CoV-2 infection. Since previous research on the circulation of SARS COV-2 in wildlife species is rather scarce, with only one study reported at the start of the SARS CoV-2 pandemic in China [33], the aim of our study was to investigate the possibility of natural infection by SARS CoV-2 of free-living and captive wild animals within their ecological niche. Therefore, In 2020/2021, we tested samples of dominant wildlife species in Croatia, the wild boar (*Sus scrofa*), red fox (*Vulpes vulpes*) and jackal (*Canis aureus moreoticus*), for the presence of SARS CoV-2 RNA and anti-SARS CoV-2 antibodies. The species were selected on the presumption that their high population density and natural habitat, possible contact with domestic animals and indirectly human (through the contaminated environment) can result in them being targets of SARS CoV-2 spillover events. Furthermore, we included yellow-legged gulls (*Larus michahellis*) in the study since they feed on garbage pits near highly populated cities. To fulfill the epidemiological data, we also tested fecal samples of zoo animals consecutively in contact with SARS CoV-2 RNA-positive humans for the presence of viral RNA.

To the best of our knowledge, this study reports the first results of investigating the possible circulation of SARS CoV-2 in selected free-ranging wildlife species outside of China and during the SARS CoV-2 s wave.

## 2. Results

Serology results within our investigation showed some differences when two different methods were used (Table 1). Six wild boars (3.9%), six red foxes (2.9%) and three jackals (4.6%) showed to be repeatedly positive for anti-SARS CoV-2 antibodies against the N protein when the ELISA test was applied (Table 1). 

Even so, samples of muscle extracts originating from foxes and jackals needed to be diluted 1:5 to avoid background effects that were present and visible in 79 tested fox samples and 16 samples originating from jackals before dilution. The optimal dilution was determined by serial dilutions of highly positive samples until the detection point showed valid repeatability and reproducibility without visible background effect, in our case with a 1:5 dilution of the initial sample.

However, all positive samples, regardless of origin, tested negative when sVNT based on the detection of anti-SARS CoV-2 antibodies for S-protein was performed. Nevertheless, four samples originating from red foxes gave weakly positive results by sVNT in the first run conducted, followed by negative results in three following repetitions. Since all applied controls gave valid results repeatedly, and the ELISA-negative samples were also negative by sVNT. No cross-reactivity with positive anti-IBV antibody sera was found. We concluded that the findings in red foxes by sVNT were negative.

All tested fecal samples originating from wild boars, red foxes, jackals and yellow-legged gulls showed to be negative for SARS CoV-2 RNA (gene encoding E protein). The same results were obtained when zoo animals were tested. The amplification of exogenous IPC RNA was positive in 97.5% of samples, indicating the absence of RT–PCR inhibitors in the majority of samples. Nine samples that were negative for IPC in the first round of real-time RT–PCR were retested in dilution 1:5, after which they proved to be positive for IPC. These nine samples were negative for the presence of SARS CoV-2 RNA.

## 3. Discussion

Based on the results of our study, we conclude that no spillover of SARS CoV-2 occurred in wild boars, red foxes and jackals in their natural habitat during the second COVID-19 wave in Croatia. The investigated gulls were all negative for viral RNA and anti-SARS CoV-2 antibodies; therefore, the passive transmission of SARS CoV-2 by gulls is excluded, and the lack of viral RNA in fecal samples of zoo animals indicates the absence of virus transmission from infected animal handlers to zoo animals during the trial. We, therefore, conclude that during our investigation SARS CoV-2 did not transmit from infected people or the environment to the tested animals even though the investigation included samples collected during the peak of the second COVID-19 infection wave in Croatia (November 2020 to February 2021. However, in 15 of 533 (2.8%) tested free-living wild animals, positive anti-SARS CoV-2 antibodies towards the N-protein were detected by ELISA. In contrast, sVNT as a confirmatory method used in this study derived negative results in all.

The estimated number of wild boars (according to the hunting bag) in Croatia varies from 45,700 to 65,000 per year and is increasing. They are the main species of interest in big game hunting within continental Croatia. Nevertheless, wild boars are recognized as reservoirs of animal pathogens and human pathogens, including causative agents of human viral diseases such as hepatitis E [37]. Since they live in families, spreading pathogens within a group is likely. When young males mature, they leave the family and can spread pathogens by their movement within a distance of even 50 km. Wild boars often visit garbage pits as feeding sites and inhabit areas nearby cities and pig farms; therefore, they are suitable for being infected by pathogens transmitted from humans, pigs and other animal species, not only through direct contact during hunting but through waste or contaminated water, as well. Our findings regarding SARS CoV-2 in wild boars agreed with previous research [23] when experimental infections of pigs resulted in no viral RNA detection in swabs. All investigated pigs were seronegative for SARS-CoV-2 antibodies by ELISA. Nevertheless, a recent study confirmed the possibility of experimental infection of domestic pigs by SARS CoV-2 [42]. Deng et al. (2020) included in their serological survey one wild boar sera with a negative result also gained by ELISA [33]. Even though our findings in general correspondent with previous reports [23,33] showing a low-risk for wild boars to be naturally infected by SARS CoV-like viruses, the finding of positive ELISA results in six wild boars (3.9%) is an exception. The same samples were negative by sVNT, indicating false-positive ELISA results due to unspecific reactions possibly due to lower sample quality. Concerning the viral RNA, the same results were observed in investigated wild boars within our study. Hence, evidence of wild boars having a role as reservoirs of disease is currently excluded. For better understanding, further investigation and optimization of serological methods when wildlife samples are used is warranted.

According to the data of the Ministry of Agriculture, the number of red foxes in Croatia is approximately 15,000. As wild boars, the red fox lives in families and smaller groups. They belong to the order *Carnivora* and feed mostly on small rodents but also on rabbits that are susceptible to SARS CoV-2 [43], reptiles and, depending on the size, even young ungulates. The red fox is a popular hunting trophy but also a reservoir of human and animal pathogens [44]. Nowadays, the red fox is entering the outskirts of cities in search of food and possible contacts with people are not excluded. Since the red fox has shown a potential to be susceptible to SARS CoV-2 [45], we included it within our study. Jackals mostly live in pairs and are highly territorial. Their population size is estimated to be around 10,000. However, their number, as well as the localities they inhabit, are rapidly increasing in Croatia. Jackals are interesting as potential reservoirs of zoonotic diseases since they are omnivores, predators and scavengers. Moreover, as members of the Canidae family, the red fox and jackal are previously found to be carriers of coronaviruses [44,46]. However, the only available study considering the in vivo susceptibility of wild Canids towards SARS CoV-2 reported negative results by serological testing of 89 foxes and one jackal in China at the beginning of the COVID-19 pandemic [33]. According to the results of experimental infections of pet dogs, their susceptibility towards SARS CoV-2 is low [23]. Nevertheless, naturally infected and serologically positive pet dogs of infected owners have been reported [47,48]. In our study, no viral RNA was detected in fecal swabs of foxes or jackals throughout the investigated period, excluding their contact with SARS CoV-2 and as possible reservoirs and virus shedders. Nonetheless, positive samples have been observed by ELISA in both, but as in wild boars, they were not confirmed by sVNT. Moreover, four of six ELISA-positive samples originating from red foxes were weak positive by sVNT in the first run. Still, the samples turned out to be negative based on the absence of repeatability and reproducibility. We presume that the false-positive results are a consequence of the specificity of the samples being muscle extracts, not sera or plasma as recommended by the manufacturer. Nevertheless, our approach in investigating muscle extracts for the presence of specific antibodies has been previously successfully applied for assessing the efficiency of the rabies eradication program in red foxes [49].

As expected, all tested yellow-legged gulls were negative for the presence of SARS CoV-2 RNA in fecal swabs, and no serological evidence of contact with the virus was detected. We tested gulls that feed on a garbage pit nearby Zagreb that, at the time of sampling, was the most affected COVID-19 location in Croatia. Therefore, our aim was to investigate the possibility of passive transmission of the virus through potentially infected human waste. Fortunately, no sign of passive or active transmission was found.

Zoo animals were tested for possible virus shedding due to their consecutive contact with zoo workers infected by SARS CoV-2. No viral RNA was found in in-contact animals regardless of species, contributing to the hypothesis that human to animal transmission is uncommon. However, serological testing that was not carried out in our study to avoid stress and direct contact would be needed for a final conclusion.

Our results also signify the need for further optimization of known serological protocols before massive animal testing. Our study included two different serological methods, both with high specificity and sensitivity relying on the detection of anti-SARS CoV-2 antibodies towards the main antigens of N and S proteins of coronaviruses, as previously recommended [50]. However, in 15 (2.8%) samples of the selected wild animal species, the methods used derived different results. Even though limited or even a lack of producing detectable neutralizing antibodies towards the S-protein were previously reported in humans and dogs [48,50], within this study, we concluded that the results observed by sVNT are conclusive since neutralizing assays are considered to be a golden standard for coronavirus serological testing.

The ELISA that targets antibodies against the N-protein of SARS CoV-2 has been validated according to different animal species (dog, cat, cattle, horse, goat, sheep, bat, agouti, opossum, ocelot, jaguar, camel and howler monkey) and for cross-reactions with other coronaviruses (avian and pig). Similar results were obtained by a double antigen ELISA based on detecting anti-SARS CoV-2 antibodies toward the S-protein [33]. Both can be used for massive serological testing of different animal species. However, due to a limited number of serologically positive animal sera, further optimization is needed according to different animal species. For our trial, we also included sera containing avian anti-IBV antibodies and seropositive human samples. While the latter constantly showed in each run positive results, no cross-reactivity was detected with anti-IBV-antibody-positive sera when both serological methods were used. The sVNT method we used is also highly sensitive (93.8%) and specific (99.4%), according to the validation results [51]. As mentioned, we presume that the differences in ELISA results and sVNT are based on the sample choice, especially in the case of foxes and jackals when muscle extracts were used. Thus the sample features may have interfered with the results. Further investigation is needed, especially regarding optimizing protocols when wildlife samples are used since they often are of lower quality than samples undertaken from domestic animals. Optimization of sVNT protocols according to different animal samples and species would highly contribute to the fast and secure detection of anti-SARS CoV-2 antibodies in the future.

In conclusion, our findings contribute to the confirmation that the dominant species investigated within this study, the wild boars, red foxes, jackals and yellow-legged gulls, show no evidence of contact with SARS CoV-2 or spillover events. Furthermore, gulls probably do not present a public health risk since passive viral transmission has not been confirmed in our study. Moreover, our results confirm that no viral transmission from positive SARS CoV-2 RNA zoo workers to in-contact zoo animals appeared within the investigated period, confirming that interspecies viral transmission is not a likely event. However, the high mutation rate of coronaviruses can cause changes in host affinity, allowing interspecies transmission. Only recently, a haplotypic variation of the viral S protein was described [11]. Proteins needed for viral cell entry that allow replication of the virus under laboratory conditions have been identified in pig cell cultures [31,52]. Moreover, Zhai et al. in 2020 reported that pigs and dogs, and even cattle and sheep appear to have ACE2 proteins capable of acting as a receptor for viral entry, even though expressed in relatively low levels in their respiratory tract [32]. Furthermore, the evidence of human-to-mink-to-human SARS CoV-2 transmission in natural conditions and the variety of viral strains found accentuates the need to further investigate potential SARS CoV-2 carriers among animals [53,54]. Consequently, new SARS CoV-2 mutations in the future may present a challenge not only for public health but also as a health risk for wildlife and domestic animals. They can cause devastating outcomes within the environment. Therefore, targeted monitoring of wildlife regarding SARS-CoV-2, especially those species that have proven to be potential reservoirs of SARS CoV-2, such as members of the Mustelidae family [26,53], is highly needed for a better epidemiological understanding of COVID-19 infection, as of other coronavirus infections. Furthermore, risk assessments, developing preparedness measures, and implementing wildlife and public health protection measures are highly important. This is possible only through the One Health platform [55].

## 4. Materials and Methods

### 4.1. Sample Collection and Preparation

Samples of wild boars (*Sus scrofa*; blood, feces), red foxes (*Vulpes vulpes*; muscle extracts, feces) and European jackals (*Canis aureus moreoticus*; muscle extracts, feces) were collected from June to the end of December 2020, according to ongoing annual monitoring programs (African and Classical swine fever, Rabies program) of the Croatian Ministry of Agriculture, Veterinary and Food Safety Directorate. Samples (blood, cloacal swabs) of yellow-legged gulls (*Larus michahellis*) were collected in November 2020 within a municipality project ‘Investigating viral and bacterial diseases of gulls feeding on a garbage pit in Zagreb’ supported by the Zagreb city Holding Center (ZGOS Ltd., Zagreb, Croatia).

All samples were randomly chosen to take into account the sample quality and geographical origin. Zoo animals included in the study (Table 1) were tested for the presence of SARS CoV-2 RNA in fecal samples collected the same day (10 February 2021). The only touchstone was to involve mammals that have been in contact with SARS CoV-2 RNA-positive handlers. The information of the zoo workers that were SARS CoV-2 RNA-positive (ten of them) from September 2020 until March 2021 was kindly shared through a signed letter of agreement. All of them except one were anti-SARS CoV-2 antibody positive and in daily contact with the animals included in the study. The antibody-negative worker was just recently infected in regards to the date of the sampling of animals.

In total, 153 wild boars (153 blood and 93 fecal samples), 204 red foxes (204 muscle extracts and 94 fecal samples), 65 jackals (65 muscle extracts and 33 fecal samples) and 111 yellow-legged gulls (111 blood and 111 cloacal swabs) were tested for the presence of anti-SARS CoV-2 antibodies and for the presence of SARS CoV-2 RNA. In contrast, fecal samples of 32 animal species that were collected from the Zagreb Zoo were tested for the presence of SARS CoV-2 RNA.

Blood samples of wild boars were collected directly from the heart of shot wild boar carcasses by educated hunters. Yellow-legged gulls were captured using cannon nets on a Zagreb city garbage pit (45.45 N, 16.01 E). They were handled by trained biologists, and blood samples (2 mL) were taken from v. ulnaris. Sera were prepared from blood samples by centrifugation at 1000× *g* through 15 min and stored at −20 °C until further testing. Most of the captured gulls were in their first calendar year (N = 71).

Regarding fox and jackals, muscle samples were collected to detect anti-SARS CoV-2 antibodies. A segment of m. femoralis (approximately 5 cm × 7 cm) was taken from their carcasses by trained pathology technicians at the Croatian Veterinary Institute Pathology laboratories. The muscles were placed in polypropylene containers (security screw cup containers, 120 mL, DeltaLab), frozen for four days and then placed at 4 °C for 3–5 days. From each piece of muscle, approximately 200–500 µL of the muscle extract was collected, centrifuged at 220× *g* for 10 min and stored at −20 °C before analysis. On the day of testing, all samples were heat-treated at 56 °C for 30 min and centrifuged (220× *g*).

Fecal samples and cloacal swabs were collected from individual animals (same group of animals as for blood sampling; samples taken directly from rectum/cloaca) except for zoo animal species where 25 of 32 samples were pooled fecal samples (2–10 animals of the same species per enclosure) (Table 2). 

Fecal samples were resuspended in phosphate-buffered saline (PBS, pH 7.4) to obtain 20% *w/v* fecal suspensions, which were then vortexed for 30 s and centrifuged for 3 min at 14,000× *g*. Supernatants were further used as starting material for RNA extraction. Dry cloacal swabs were resuspended in 1 mL of PBS and further processed by the same procedure described for fecal samples.

### 4.2. Detection of Specific Anti-SARS CoV-2 Antibodies by Enzyme-Linked Immunosorbent Assay (ELISA)

A commercially available enzyme-linked immunosorbent assay (ELISA) kit (ID Screen SARS CoV-2 double antigen multispecies; ID.Vet, France) was used for the detection of specific antibodies against the nucleocapsid antigen (N) of SARS CoV-2 according to the manufacturer’s instructions. Samples showing the sample to positive ratio (S/P) percentage greater than or equal to 60% are considered as positive, and those with the S/P % less or equal to 50% are considered as negative. The optical densities (OD) of each sample were detected at 450 nm by a spectrophotometer (TECAN, Männedorf, Switzerland). Apart from the positive controls provided in the kit, we used the additional SARS CoV-2 antibody-positive and negative human sera samples (kindly provided by the University Hospital for Infectious Diseases “Dr. Fran Mihaljević”, Zagreb Croatia). For the negative control, an additional avian serum containing anti-infectious bronchitis virus antibodies (anti-IBV) from our collection was added.

### 4.3. Detection of SARS-CoV-2 Neutralizing Antibodies by a Surrogate Virus Neutralization Test (sVNT)

The cPASS SARS-CoV-2 neutralization antibody detection kit (GenScript Biotech, Amsterdam, Netherlands) was used to investigate the presence of specific anti-SARS-CoV-2 neutralizing antibodies (NAbs) against the S-protein in serum and muscle extract samples in a species and isotype-independent manner by blocking the interaction between the receptor-binding domains (RBD) of the viral spike glycoprotein with the ACE2 cell surface receptor. The procedure is based upon mimicking the virus–host interaction by direct protein–protein interaction following the same manner as conventional virus neutralization tests. This test was commercialized upon the establishment of sVNT, which detects specific anti-SARS CoV-2 NAbs without the need to use live viruses or cells, by the Singapore group of scientists [51]. The test was used on all samples that tested positive on ELISA (N protein) and randomly chosen ELISA-negative samples. The protocol was applied according to the manufacturers’ instructions. Spectrophotometry was conducted at 450 nm in a plate reader (TECAN, Switzerland). If present in the sample, neutralizing antibodies block the reaction of HRP-RBD with hACE2. The absorbance of the sample is inversely dependent on the titer of the anti-SARS-CoV-2 neutralizing antibodies in tested samples. For controls, we used the same sera samples as described for ELISA.

### 4.4. RNA Extraction and SARS CoV-2 Detection by Real-Time RT–PCR

Viral RNA was extracted from 200 μL supernatant of prepared fecal/cloacal swab samples using a MagMAX core kit (Thermo Fisher Scientific, Waltham, MA, USA) on a KingFisherTM Flex purification system (Thermo Fisher Scientific, Waltham, MA, USA) according to the manufacturer’s instructions. The exogenous internal positive control (IPC) RNA, Xeno™ RNA control (Thermo Fisher Scientific, Waltham, MA, USA), was added to each sample (2 µL) to monitor the presence of PCR inhibitors. RNA extracts were stored at −80 °C until used. To detect SARS CoV-2 RNA, samples were tested with an “E-gene Sarbeco FAM” protocol [56]. Real-time RT–PCR reactions were performed using qScript XLT One-Step RT–qPCR ToughMix (Quanta Bio, Beverly, MA, USA) in reaction conditions as prescribed by the manufacturer. The PCR was conducted by a Rotorgene Q (Qiagen, Hilden, Germany) and QIAquant 96 5plex (Qiagen, Hilden, Germany). In each test, we used synthetic ssRNA fragments of SARS-CoV-2 (EURM-019; JRC, European Commission) as a positive control (kindly provided by the University Hospital for Infectious Diseases “Dr. Fran Mihaljević”, Zagreb) and no template control (NTC) was used to monitor eventual nucleic acid contamination. The exogenous IPC RNA was detected in a separate reaction using VetMAXTM-Plus one-step RT–PCR kit (Thermo Fisher Scientific, Waltham, MA, USA) and the VetMAX™ Xeno™ internal positive control (IPC) VIC™ assay (Thermo Fisher Scientific, Waltham, MA, USA) by following the manufacturer’s instructions. These reactions were run on the same instruments as for SARS CoV-2 detection.

## 5. Conclusions

Based on the gained results, we conclude that during the investigated period (June 2020-March 2021), no SARS CoV-2 virus transmission from infected people or potentially contaminated environment to free-living wild animals included in the study occurred. Our conclusion also refers to in-contact zoo animals regardless of the fact that we collected samples during the ongoing second COVID-19 wave in Croatia when the animals were in contact with infected zoo workers. This demonstrates that the animal–human transmission of SARS CoV-2 is uncommon. Gulls were included in the study as potential passive transmitters of the virus and, as such, were excluded from this role. However, further investigation is needed and targeted monitoring of animals and optimization of serological protocols, especially when wildlife sampling is conducted.

## Figures and Tables

**Table 1:** Results of serological testing (ELISA and sVNT) of free-living wild animals.

	Number of Tested Animals	Sampling Period within 2020	Testing Results (absolute Numbers and %)	
			ELISA	sVNT
Yellow-legged gulls (*Larus michahellis*)	111	November	Neg.	Neg.
2. Wild boars (*Sus scrofa*)	153	June–December	6 (3.9%; 95% CI = 1.5%–8.3%)	Neg.
3. Red foxes (*Vulpes vulpes*)	204	June–November	6 (2.9%; 95% CI=1.0%–6.2%)	Neg.
4. Jackals (*Canis aureus moreoticus*)	65	June–October	3 (4.6%; 95% CI = 0.9%–12.9%)	Neg.
∑	533	June–December	15 (2.8%; 95% CI = 1.7%–4.5%)	Neg.

**Table 2:** Animal species tested in captivity (Zagreb Zoo).

	Species	Age (Years)	Gender (M—Male, F—Female), Mixed—Pool
1	Big hairy armadillo (*Chetophractus villosus*)	3–15	Pool
2	Southern three-banded armadillo (*Tolypeutes matacus*)	1–3	Pool
3	Tufted capuchin (*Sapajus apella paella*)	0.5–19	Pool
4	Black howler (*Alluata caraya*)	6–12	Pool
5	Mantled guereza (*Colobus guereza*)	6–14	Pool
6	Northern plains gray langur (*Semnopithecus entellus*)	0.8–19	Pool
7	Lar gibbon (*Hylobates lar*)	0.8–21	Pool
8	Chimpanzee (*Pan troglodytes*)	0.9–21	Pool
9	Crowned lemur (*Eulemur coronatus*)	0.9–4	Pool
10	Ring-tailed lemur (*Lemur catta*)	7–23	Pool
11	Black-and-white ruffed lemur (*Varecia variegata variegate*)	0.8–12	Pool
12	Egyptian fruit bat (*Rousettus aegyptiacus*)	n.d.	Pool
13	Common noctule (*Nyctalus noctula*)	n.d.	F
14	Nathusius’s pipistrelle (*Pipistrellus nathusi*)	n.d.	M
15	Serval (*Laptailurus serval*)	18	F
16	Lynx (*Lynx lynks*)	22	F
17	African Lion (*Panthera leo*)	5–16	Pool
18	Chinese leopard (*Panthera pardus japonensis*)	2–5	Pool
19	Common dwarf mongoose (*Helogale parvula*)	0.4–4	Pool
20	Meerkat (*Suricata suricatta*)	0.5–6	Pool
21	European wolf (*Canis lupus*)	3–7	Pool
22	Brown bear (*Ursus arctos*)	33	F
23	Asian small-clawed otter (*Aonyx cinereus*)	9–11	Pool
24	Red panda (*Ailurus fulgenis*)	6.5	F
25	Göttingen dwarf pig (*Sus scrofa domestic*)	7	F
26	Collared peccary (*Pecari tajacu*)	7–21	Pool
27	Pygmy hippopotamus (*Hexaprotodon liberiensis*)	6–21	Pool
28	Bactrian camel (*Camelus bactrianus*)	11–18	Pool
29	Alpaca *(Lama pacos*)	16	Pool
30	Llama (*Lama glama*)	3–5	Pool
31	American pygmy goat (*Capra hircus*)	8–9	Pool
32	Sheep (*Ovis aries domestic*)	4–9	Pool

## Data Availability

The datasets used and/or analyzed within the frame of the study can be provided by the corresponding author (ilojkic@veinst.hr), as well as the first (jemersic@veinst.hr) and last (brnic@veinst.hr) authors upon reasonable request.
